# Supplementary material for: Associations between COVID-19-Related Digital Health Literacy and Online Information-Seeking Behavior among Portuguese University Students
Source: Int J Environ Res Public Health. 2020 Dec 2;17(23):8987. doi: 10.3390/ijerph17238987 (PMC7730120; doi:10.3390/ijerph17238987)
Supplement: Supplementary file 1 [file ijerph-17-08987-s001.pdf]

Supplementary table Associations between sources of information and DHL related with COVID-19

	DHL related with COVID-19 OR (95%CI)			
	Information Searching	Adding self-generated content	Evaluating reliability	Determining relevance
Weeks of data collection				
Week 1	ref	ref	ref	ref
Week 2	1.0 (0.7; 1.2)	1.0 (0.8; 1.4)	0.9 (0.7; 1.1)	1.0 (0.8; 1.3)
Week 3 and 4	0.7 (0.5; 1.1)	0.8 (0.5; 1.3)	0.7 (0.5; 1.1)	1.0 (0.7; 1.6)
Week 5 and 6	1.1 (0.9; 1.4)	0.9 (0.7; 1.2)	0.9 (0.7; 1.1)	0.8 (0.6; 1.0)
Subjective social status	1.2 (1.0; 1.4)	1.2 (1.0; 1.5)	1.2 (1.0; 1.4)	**1.3 (1.1; 1.5)**
Search engines (eg. Google, Bing, Yahoo!)	**0.7 (0.5; 0.9)**	0.8 (0.6; 1.1)	0.8 (0.6; 1.1)	0.8 (0.6; 1.1)
Websites of public bodies	1.4 (0.9; 2.1)	1.4 (0.9; 2.3)	**1.7 (1.1; 2.5)**	**1.7 (1.1; 2.5)**
Wikipedia and other online-encyclopaedias	0.8 (0.7; 1.0)	0.9 (0.7; 1.1)	**0.7 (0.6; 0.9)**	**0.7 (0.6; 0.9)**
Social media (eg. Facebook, Instagram, Twitter)	**0.7 (0.6; 0.9)**	0.8 (0.7; 1.0)	**0.7 (0.6; 0.8)**	0.9 (0.7; 1.1)
YouTube	1.0 (0.8; 1.2)	1.0 (0.8; 1,3)	0.9 (0.7; 1.1)	1.0 (0.8; 1.2)
Blogs on health topics	0.9 (0.7; 1,2)	1.0 (0.8; 1,3)	0.9 (0.7; 1,1)	0.9 (0.7; 1,1)
Guidebook-communities (eg. SNS-24)	1.1 (0.8; 1.3)	1.1 (0.8; 1.4)	0.8 (0.7; 1.1)	1.0 (0.8; 1.2)
Health portals	1.0 (0.8; 1.2)	1.1 (0.9; 1.4)	**1.3 (1.0; 1.5)**	1.1 (0.9; 1.4)
Websites of doctors or health insurance companies	1.0 (0.8; 1.3)	1.1 (0.9; 1.4)	1.1 (0.9; 1.3)	1.1 (0.9; 1.4)
News portals (eg. of newspapers, TV stations)	1.0 (0.8; 1.4)	1.0 (0.8; 1.4)	1.2 (0.9; 1.5)	1.1 (0.9; 1.5)

Unadjusted results from binary logistic regression *Odds ratio* (Confidence Interval).

Bold $p<0.05$
